# NADPH-cytochrome P450 reductase involved in the multi-resistance of *Aphis spiraecola* to imidacloprid and lambda-cyhalothrin

**DOI:** 10.3389/fphys.2026.1944804

**Published:** 2026-09-11

**Authors:** Shuo Zhang, Kang Wang, Yi You, Tong Wang, Wenrong Xian, Hua Wu, Hainan Shao, Zhiqing Ma

**Affiliations:** 1 Key Laboratory of Plant Protection Resources and Pest Management of Ministry of Education, College of Plant Protection, Northwest A&F University, Xianyang, Shaanxi, China; 2 Provincial Center for Bio-Pesticide Engineering, Northwest A&F University, Xianyang, Shaanxi, China; 3 State Key Laboratory of Plateau Ecology and Agriculture, Qinghai Academy of Agriculture and Forestry Sciences, Qinghai University, Xining, China

**Keywords:** *Aphis spiraecola*, imidacloprid, lambda-cyhalothrin, multi-resistance, NADPH-cytochrome P450 reductase

## Abstract

NADPH-cytochrome P450 reductase (CPR) serves as the obligate electron donor for cytochrome P450 enzymes and is essential for xenobiotic metabolism and detoxification. However, its involvement in insecticide resistance of *Aphis spiraecola* remains unclear. In this study, the open reading frame (ORF) of the *A. spiraecola CPR* gene was cloned and characterized. qRT-PCR analysis revealed that *AsCPR* was most abundantly expressed in apterous adults of the susceptible (SS), imidacloprid-resistant (IM-R) and lambda-cyhalothrin-resistant (LC-R) strains, with higher transcript levels in the hemolymph and midgut. In different strains, *AsCPR* expression was stably induced by both imidacloprid and lambda-cyhalothrin, with induction levels ranging from 1.21 to 4.9-fold. Furthermore, SPc-mediated RNAi of *AsCPR* achieved silencing efficiencies exceeding 40% at 12 h post-injection in both resistant strains. This knockdown significantly reduced P450 enzyme activity by 18.4% in the IM-R strain and 16.6% in the LC-R strain. Accordingly, the sensitivities of the IM-R strain to imidacloprid and the LC-R strain to lambda-cyhalothrin were significantly increased, with LC_50_ values decreasing by 57.6% and 49.8%, respectively, compared with the ds*GFP* control group. Finally, the recombinant AsCPR protein was expressed *in vitro*, and its activity was assessed. The results indicate that AsCPR plays a key role in the development of multi-resistance of *A. spiraecola* to pyrethroid and neonicotinoid insecticides.

## 1 Introduction

The aphid *Aphis spiraecola* is a globally distributed pest, which can damage a variety of plants including apple, pear and spirea (Yan et al., 2018; You et al., 2026). This insect causes significant losses to host plants through direct feeding damage and indirect virus transmission (Cho et al., 1997). For decades, chemical control has long been regarded as the primary management strategy against *A. spiraecola*, with neonicotinoid and pyrethroid insecticides as the most frequently used synthetic insecticides (Wang K. et al., 2025). However, the prolonged and intensive application of these insecticides has led to the development of severe resistance in *A. spiraecola* (Tang et al., 2025; Wang K. et al., 2025).

Insecticide resistance in arthropod pests is underpinned by multiple molecular mechanisms, among which metabolic resistance resulting from the elevated expression of detoxification enzymes represents a particularly common route (Li et al., 2007; Feyereisen et al., 2015). Cytochrome P450 monooxygenases (P450s), a superfamily of heme-thiolate proteins, play indispensable roles in the oxidative metabolism of both endogenous substrates and xenobiotic compounds (Li et al., 2007; Schuler, 2011). Notably, the enhanced detoxification capacity of P450s has been implicated in conferring resistance across diverse insecticide classes (Nauen et al., 2022).

The NADPH-cytochrome P450 reductase (CPR) is the sole physiological electron donor for the P450 system and is critical for P450-mediated detoxification of insecticides (Wang et al., 1997; Feyereisen, 1999). P450s rely primarily on CPR to acquire electrons from NADPH. These electrons are sequentially relayed from the FAD domain to the FMN domain, ultimately reaching the P450 heme-binding site to initiate the catalytic reaction (Pandey and Flück, 2013). Multiple studies had shown that knockdown of the *CPR* gene significantly enhanced pest susceptibility to insecticides. The reduced *CPR* expression in *Acyrthosiphon pisum* increased susceptibility to chlorpyrifos, imidacloprid and beta-cypermethrin (Qiao et al., 2022). Similarly, knockdown of *DcCPR* enhanced the sensitivity of *Diaphorina citri* against imidacloprid and thiamethoxam (Yuan et al., 2021). Indeed, P450s had been unequivocally implicated in resistance to both imidacloprid and lambda-cyhalothrin in *A. spiraecola* (Tang et al., 2025; Wang K. et al., 2025). However, the role of AsCPR in driving insecticide resistance in *A. spiraecola* via the P450/CPR system remains elusive.

In this study, the full-length sequence of *AsCPR* was identified and characterized. The spatiotemporal expression pattern of *AsCPR* and its response to induction by imidacloprid and lambda-cyhalothrin were investigated using qRT-PCR, respectively. The role of AsCPR in P450-mediated resistance to imidacloprid and lambda-cyhalothrin was evaluated by RNA interference (RNAi) and further elucidated via *in vitro* functional protein expression.

## 2 Materials and methods

### 2.1 Insects and insecticides

The susceptible strain (SS) of *A. spiraecola* was originally collected in 2020 from the economic tree arboretum of Northwest A&F University (Yangling, China), and had been continuously maintained in the laboratory without any insecticide exposure. The imidacloprid-resistant (IM-R) and lambda-cyhalothrin-resistant (LC-R) strains were initially collected in 2020 from Qianxian, Shaanxi Province (Wang K. et al., 2025), and established through continuous selection with imidacloprid and lambda-cyhalothrin, respectively. During this process, the aphids were screened every four generations, and the optimal insecticide concentration (causing 50–60% mortality) for each screening was predetermined prior to selection. All strains were reared on apple seedlings in the greenhouse under the controlled environmental conditions: 28 ± 1 °C, relative humidity of 50-60% and a photoperiod of 16-h light/8-h dark.

Imidacloprid (97%) was supplied by Jiangsu Changlong Agrochemical Co., Ltd. (Jiangsu, China). Lambda-cyhalothrin (98%) was supplied by Shanxi Qixing Pesticide Co., Ltd. (Shanxi, China). The nanocarrier SPc was a gift from Dr. Shuo Yan (China Agricultural University).

### 2.2 Toxicity bioassay

The sensitivity of imidacloprid and lambda-cyhalothrin was determined according to the leaf-dipping method described by Wang K. et al. (2025). Briefly, the technical insecticides were initially dissolved in acetone and subsequently serially diluted with 0.1% (v/v) Tween 80 solution to prepare five gradient concentrations. Apple leaves infested with 50-60 apterous adult aphids were dipped into the corresponding insecticide solution for 10 s, and the residual liquid was gently absorbed from the leaf surface using dry filter paper. After treatment, the aphids were transferred to petri dishes containing moist filter paper and maintained under controlled conditions described above. Control groups received only the 0.1% (v/v) Tween 80 solution. Each concentration was tested with three independent biological replicates. Mortality was assessed after 24 h post-exposure. Aphids that showed no response to gentle touching with a fine brush were considered dead.

### 2.3 RNA extraction and cDNA synthesis

Total RNA was extracted from 2 mg of aphids or tissues using the SteadyPure Quick RNA Extraction Kit (Accurate Biology, Changsha, China). The RNA concentrations were quantified using a NanoDrop spectrophotometer (Thermo Fisher, USA), and the integrity was verified by 1% agarose gel electrophoresis. First-strand cDNA was synthesized using the HiScript II 1st Strand cDNA Synthesis Kit (+gDNA wiper) (Vazyme, Nanjing, China).

### 2.4 Bioinformatic analysis and phylogenetic tree construction of *AsCPR*

The *AsCPR* sequence was identified from the transcriptome of *A. spiraecola* and subsequently validated by RT-PCR (**Supplementary Table 1**). The theoretical isoelectric point (pI) and molecular weight (MW) of AsCPR were predicted using ExPASy (https://www.expasy.org/). Transmembrane helices were forecasted with DeepTMHMM-1.0 (https://services.healthtech.dtu.dk/services/DeepTMHMM-1.0/), and signal peptides were analyzed using SignalP-6.0 (https://services.healthtech.dtu.dk/services/SignalP-6.0/). Functional domains and catalytic residues were annotated via the Conserved Domain Database (CDD) (www.ncbi.nlm.nih.gov/Structure/cdd/cdd.shtml). A phylogenetic tree was constructed based on the amino acid sequences of known insect CPRs using the neighbor-joining method in MEGA 11.0 with bootstrap values derived from 1000 replicates. The resulting tree was visualized using the iTOL (http://itol.embl.de).

### 2.5 Quantitative real-time PCR analysis

The relative expression levels of the *AsCPR* gene were assessed across different developmental stages (first to fourth instar nymphs and apterous adults) and various tissues (head, cuticle, hemolymph, embryo and midgut) in the SS, IM-R and LC-R strains. Gene-specific primers of *AsCPR* and the housekeeping gene *β*-actin were designed using Primer-BLAST (https://www.ncbi.nlm.nih.gov/tools/primer-blast) (**Supplementary Table 1**). qRT-PCR was carried out using FastStart™ SYBR Green I Master Mix (Yeasen, Shanghai, China) on CFX96 Touch instrument (CFX96 Touch, BIORAD, USA). The 20 μL reaction mixture consisted of 1 μL cDNA, 10 μL Hieff^®^ qPCR SYBR Green Master Mix (Yeasen, Shanghai, China), 0.4 μL forward primer, 0.4 μL reverse primer and 8.2 μL RNase-free ddH_2_O. The amplification program included an initial denaturation at 95 °C for 5 min, followed by 37 cycles of 95 °C for 10 s and 60 °C for 30 s, and finally performed melting curve analysis at 65-95 °C. Each treatment was performed with three biological replicates and three technical replicates. The 2^-ΔΔCt^ method was applied to analyze the relative expression levels of the genes (Livak and Schmittgen, 2001).

### 2.6 Determination of *AsCPR* expression level after induction

Apterous adults of the SS, IM-R and LC-R strains were exposed to imidacloprid and lambda-cyhalothrin at their respective LC_30_ concentrations (The LC_30_ values of imidacloprid against the SS and IM-R strains were approximately 0.5 and 200 mg L^-^¹; The LC_30_ values of lambda-cyhalothrin against the SS and LC-R strains were approximately 0.4 and 2800 mg L^-^¹.) for 0, 6, 12, 24, 48 and 72 h, respectively. Twenty surviving aphids were collected for qRT-PCR analysis, and an aqueous solution containing 0.1% (v/v) Tween-80 served as the control. Three biological replicates were conducted for each treatment.

### 2.7 RNA interference

The interference fragments of the *AsCPR* and green fluorescent protein (*GFP*) gene were designed using the SnapDragon dsRNA design tool (https://www.flyrnai.org/snapdragon). Double-stranded RNAs (dsRNAs) were synthesized using the TranscriptAid T7 High Yield Transcription Kit (Thermo Fisher, USA) following the manufacturer’s instructions. The synthesized dsRNA was verified by 1% agarose gel electrophoresis, and its concentration was determined using a NanoDrop spectrophotometer (Thermo Fisher, USA). The star polymer SPc, which possessed the dual capabilities of stabilizing dsRNA and facilitating its cellular internalization and endosomal escape, was mixed with target dsRNA at a 1:1 mass ratio with 1% alkyl polyglucoside (APG) surfactant, resulting in a final dsRNA concentration of 500 ng μL-1 (Ma et al., 2022). For each treatment, 100 ng of dsRNA was microinjected into the abdominal region of *A. spiraecola* using a programmable microinjector (Drummond, USA). Following injection, aphids were maintained for > 30 min to allow complete absorption before transfer to apple seedlings. The control treatments received identical preparations containing ds*GFP* instead of ds*AsCPR*. The aphids were collected at different time points for qRT-PCR analysis. Three biological replicates were conducted for each treatment. Bioassays were performed at 12 h post-injection (peak RNAi efficiency) following the method in Section 2.2.

### 2.8 Determination of P450 activity after *AsCPR* knockdown

At 12 h post-RNAi treatment, 50 apterous adults were collected for P450 activity assay, with three biological replicates and three technical replicates per treatment. P450 activity was measured according to the method described by Wang et al. (2014). Samples were homogenized in 800 μL of 0.1 M phosphate-buffered saline (PBS, pH 7.4, 1 mM EDTA, 1 mM PTU, 0.1 mM DTT, 1 mM PMSF and 10% glycerol). The homogenate was then centrifuged at 15,000 rpm for 20 min at 4 °C, and the supernatant was collected for subsequent assays. p-Nitroanisole (PNOD) was diluted to 4 mM with PBS buffer pre-warmed to 37 °C. The reaction was initiated by sequentially adding 90 μL of p-nitroanisole solution, 90 μL of enzyme solution, and 20 μL of 9.6 mM NADPH into a 96-well microplate. The reaction mixture was incubated at 30 °C for 40 min, and the absorbance was measured at 400 nm using a Sunrise microplate reader (Tecan, China). A negative control was prepared by replacing the enzyme solution with buffer and omitting NADPH. Total protein content was determined using a Bradford Protein Assay Kit (Sangon, Shanghai, China). P450 activity was quantified based on a standard curve of p-nitrophenol concentration against absorbance at 400 nm.

### 2.9 Heterologous expression and purification of recombinant AsCPR

The encoding fragment of *AsCPR* was amplified with gene-specific primers (**Supplementary Table 1**) and cloned into the vector pET30a (+) (Novagen, Darmstadt, Germany) using a one-step cloning kit (Vazyme, Nanjing, China). Subsequently, the recombinant plasmid was transformed into *Escherichia coli* BL21 (DE3) competent cells. A single positive colony confirmed by sequencing was inoculated into LB liquid medium containing 100 μg mL^-1^ kanamycin and cultured at 37 °C until the OD_600_ reached 0.6. Protein expression was induced by adding isopropyl *β*-d-1-thiogalactopyranoside (IPTG) to a final concentration of 0.4 mM, and the culture was further incubated at 16 °C and 180 rpm for 48 h. Cells were harvested by centrifugation at 8000 rpm for 10 min at 4 °C, resuspended in lysis buffer (20 mM Tris-HCl, pH 7.4; 5% glycerol, 1 mM PMSF), and disrupted via ultrasonication. Then the lysate was centrifuged at 12,000 rpm for 30 min at 4 °C, and the supernatant was collected for subsequent purification. The target protein was purified using a 10 mL Ni²^+^-NTA resin (Sangon, Shanghai, China) and eluted with using 200 mM imidazole. The purity of the recombinant protein was analyzed by sodium dodecyl sulfate-polyacrylamide gel electrophoresis (SDS-PAGE) and western blotting (WB), and its concentration was quantified with a Bradford Protein Assay Kit (Coolaber, Beijing, China).

### 2.10 *In vitro* enzymatic activity assay of recombinant AsCPR protein

The *in vitro* enzymatic activity of recombinant AsCPR protein was assayed according to a previously described method (Zhao et al., 2018) with brief modifications. All reactions were conducted at 25 °C in 50 mM Tris-HCl buffer (pH 7.4, 0.1 mM EDTA). To determine the kinetic parameters for NADPH, cytochrome c was added at a final concentration of 100 μM, and NADPH concentrations varied from 0 to 150 μM. The reduction of cytochrome c was monitored by measuring the increase in absorbance at 550 nm. The molar extinction coefficient of bovine heart cytochrome c was determined to be 21 mM^-^¹ cm^-^¹. Kinetic parameters were measured in the presence of 100 μM NADPH with cytochrome c concentrations ranging from 0 to 150 μM. Absorbance changes were recorded using a microplate reader (Thermo Fisher, USA). All measurements were performed with three technical replicates per concentration. Kinetic parameters were calculated with GraphPad Prism 10 software by nonlinear regression analysis based on the Michaelis-Menten model.

### 2.11 Statistical analysis

The LC_50_ values and 95% confidence intervals were determined by SPSS 26 (IBM, NY, USA). For comparative analyses, we employed Student’s *t*-test for pairwise comparisons and one-way analysis of variance (ANOVA) followed by Tukey’s HSD test for multiple group comparisons. All statistical procedures were performed using SPSS 26 with significance levels set at *P* < 0.05.

## 3 Results

### 3.1 Bioassay analysis

The susceptibility of the SS, LC-R and IM-R strains of *A. spiraecola* to imidacloprid and lambda-cyhalothrin was determined, respectively. The results showed that the IM-R strain developed a high level of resistance to imidacloprid (492.31-fold), and the LC-R strain showed a resistance ratio of 6159.71-fold to lambda-cyhalothrin (**Table 1**).

**Table 1:** Toxicity of imidacloprid and lambda-cyhalothrin in the SS, IM-R and LC-R strains of *Aphis spiraecola*.

Strains	Treatment	Slope ± SE	LC_50_ (mg L^−1^) (95% CI)	χ2 (*df*)	RR
SS	Imidacloprid	2.79 ± 0.22	0.79 (0.71-0.88)	2.38 (3)	–
IM-R		1.93 ± 0.16	387.98 (332.67-452.69)	0.58 (3)	492.31
SS	Lambda-cyhalothrin	1.78 ± 0.16	0.78 (0.67-0.92)	1.77 (3)	–
LC-R		2.34 ± 0.18	4823.06 (3574.07-6383.26)	5.36 (3)	6159.71

RR, LC_50_ of the IM-R strain/LC_50_ of the SS strain or LC_50_ of the LC-R strain/LC_50_ of the SS strain.

### 3.2 Characterization of the CPR sequence in *A. spiraecola*

**Figure 1:**
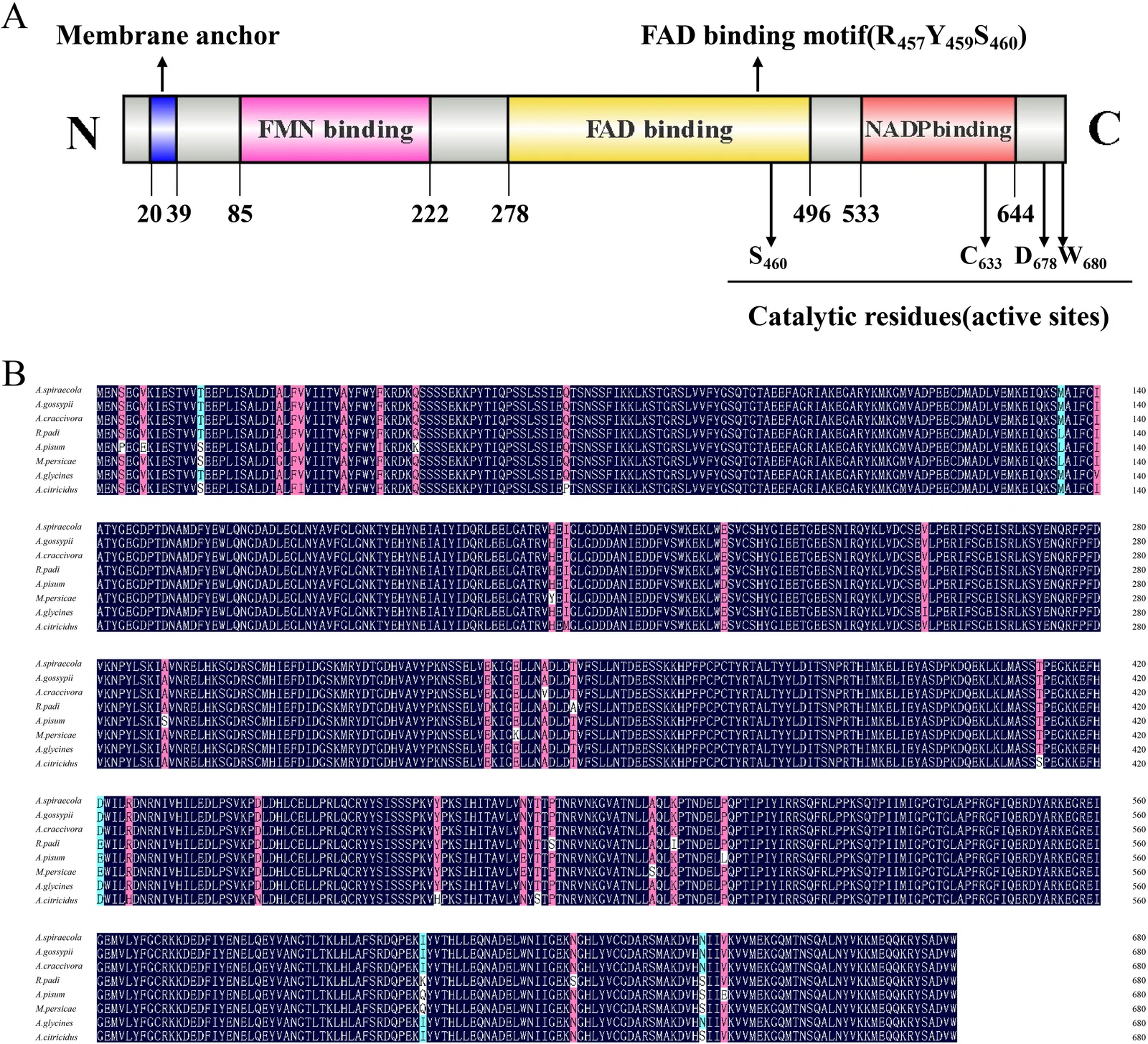
Sequence analysis of *Aphis spiraecola* CPR. **(A)** Schematic illustration of AsCPR domain structure. **(B)** Comparison of the deduced amino acid sequence of AsCPR with CPRs of *A. gossypii* (XP_050057273.1), *A. craccivora* (KAF0767109.1), *Rhopalosiphum padi* (XP_060835709.1), *Acyrthosiphon pisum* (XP_001945312.1), *Myzus persicae* (XP_022170589.1), *A. glycines* (KAE9544418.1), *A. citricidus* (AXR98634.1).

The *AsCPR* gene was 2,046 bp in length and encoded a 681-amino-acid protein, with a predicted molecular weight of 77.8 kDa and an isoelectric point (pI) of 5.27. An N-terminal membrane-anchoring region was identified in the AsCPR protein sequence, while no signal peptide was detected (**Figure 1A**). Conserved domain analysis revealed that AsCPR comprised three typical domains: an FMN-binding domain, a FAD-binding domain and an NADP-binding domain. The FAD-binding motif included three key amino acid residues: Arg457, Tyr459 and Ser460 (**Figure 1A**). The conserved catalytic residues of AsCPR were identified as Ser460, Cys633, Asp678 and Trp680 (**Figure 1A**).

### 3.3 Phylogenetic analysis of *AsCPR*

**Figure 2:**
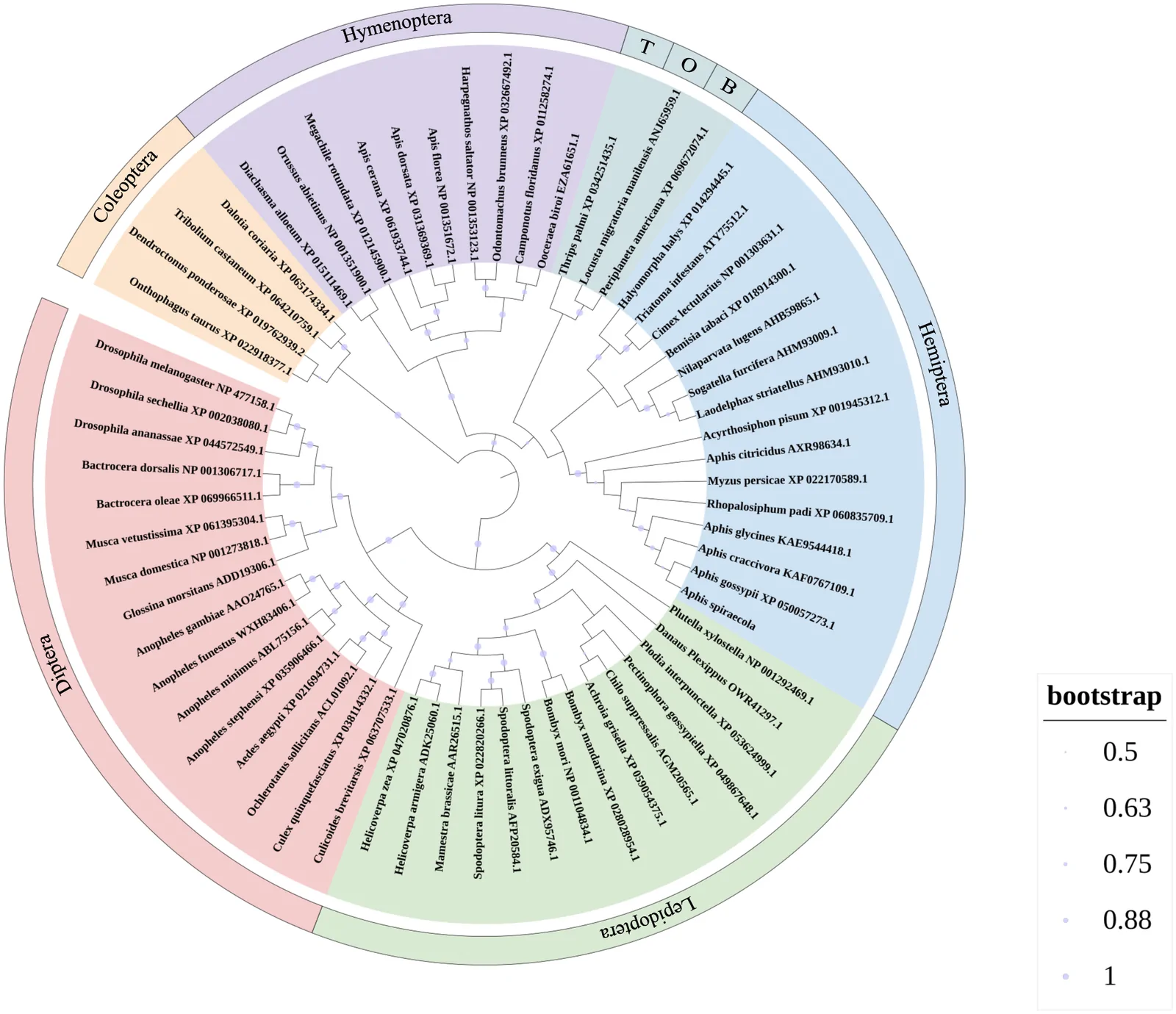
Phylogenetic relationship of *Aphis spiraecola* CPR with other insect CPRs. A phylogenetic tree of insect CPRs was constructed using the neighbor-joining method (1000 bootstrap replicates) by MEGA 11.0 software. The GenBank accession numbers of insect CPRs used for constructing of the phylogenetic tree are listed in **Supplementary Table 2**.

A phylogenetic tree was constructed based on the amino acid sequences of AsCPR and other insect CPRs. The results showed that AsCPR clustered with CPRs from other hemipteran insects into a single clade with strong bootstrap support (**Figure 2**). Multiple sequence alignment also revealed that AsCPR shared high sequence identity with CPR orthologs from other aphid species (**Figure 1B**).

### 3.4 Spatiotemporal expression pattern of *AsCPR*

**Figure 3:**
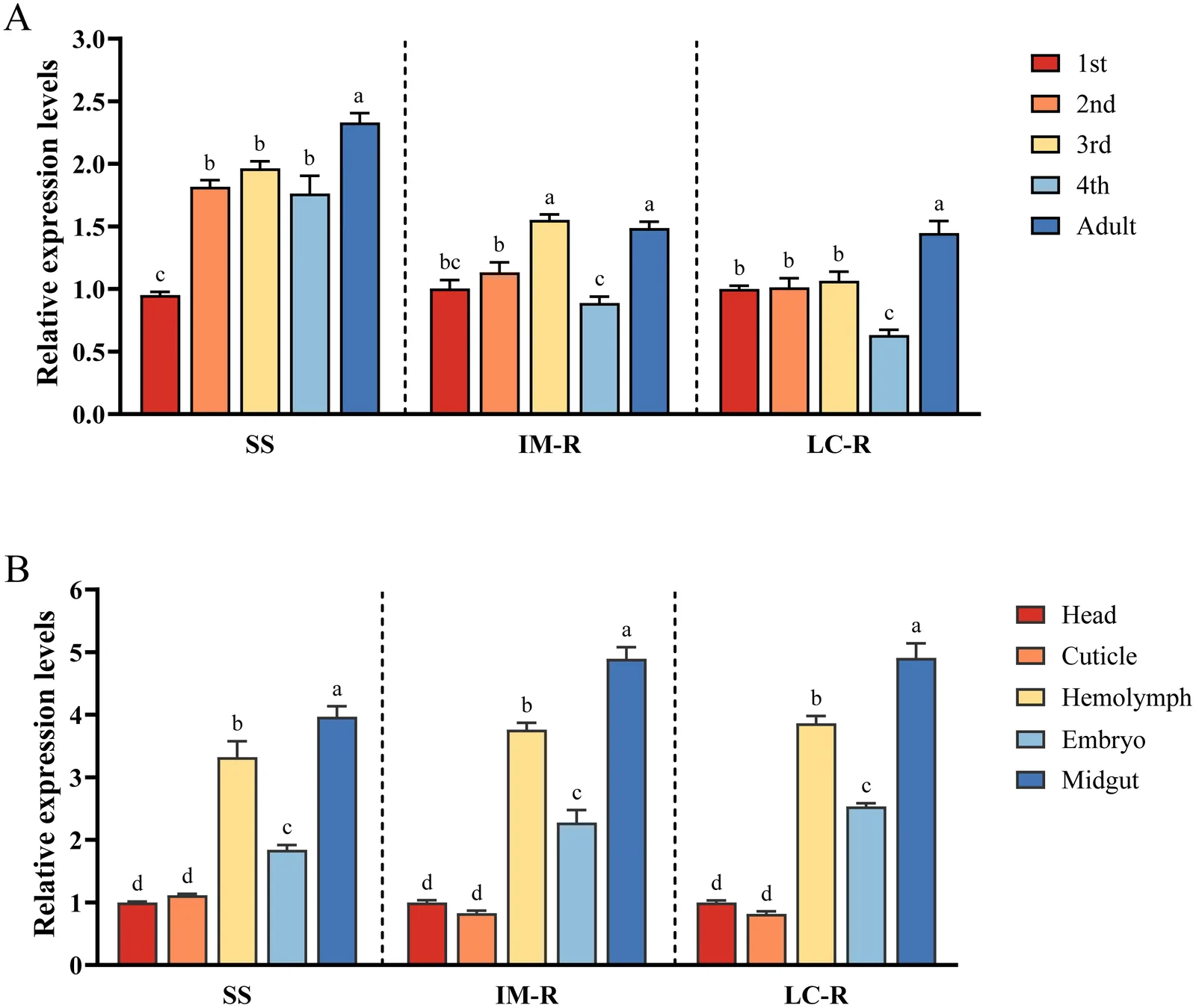
Relative expression levels of AsCPR in **(A)** different instars and **(B)** various tissues of the SS, IM-R, and LC-R strains. Data are presented as mean ± SE (*n* = 3). Different letters above the bars indicate significant differences among samples (Tukey’s HSD test, *P* < 0.05).

Developmental stage expression analysis showed that *AsCPR* was expressed across all developmental stages in the SS, IM-R and LC-R strains, with the highest transcript level detected in apterous adults (**Figure 3A**). Tissue-specific expression analysis further revealed that *AsCPR* was highly expressed in the hemolymph and midgut of all three strains, whereas relatively low expression levels were observed in the cuticle and head (**Figure 3B**).

### 3.5 Effects of imidacloprid and lambda-cyhalothrin on the expression of *AsCPR*

**Figure 4:**
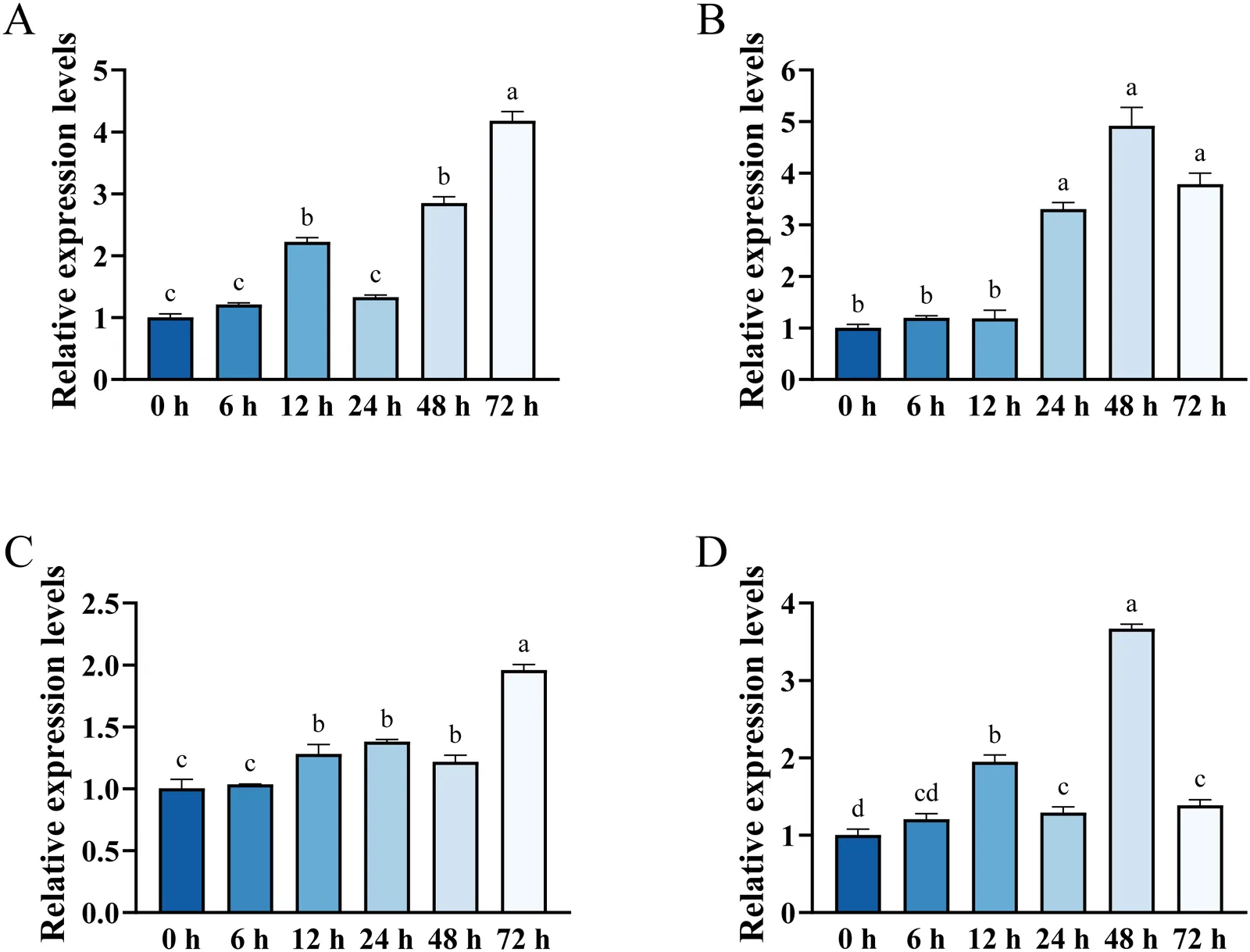
Expression levels of *AsCPR* in the SS strain of *Aphis spiraecola* under imidacloprid (LC_30_) **(A)** and lambda-cyhalothrin (LC_30_) **(B)** treatment. Expression levels of *AsCPR* in the IM-R strain under imidacloprid (LC_30_) **(C)** and in the LC-R strain under lambda-cyhalothrin (LC_30_) **(D)**. Data were shown as mean ± SE (*n* = 3). Different letters indicate a statistically significant difference among samples (Tukey’s HSD test, *P* < 0.05).

To further investigate the response patterns of *AsCPR* to imidacloprid and lambda-cyhalothrin, expression levels of *AsCPR* in the SS, IM-R and LC-R strains were quantified by qRT-PCR after exposure to LC_30_ concentrations of insecticides. In the SS strain, both insecticides significantly induced the upregulation of *AsCPR*, with imidacloprid eliciting a progressive increase that peaked at 72 h (4.2-fold) (**Figure 4A**), and lambda-cyhalothrin inducing a rapid rise that peaked at 48 h (4.9-fold) (**Figure 4B**). In the IM-R strain, imidacloprid treatment resulted in a sustained upregulation across all time points, with the maximum expression observed at 72 h (1.95-fold) (**Figure 4C**). Lambda-cyhalothrin induced a transient response of *AsCPR* in the LC-R strain, peaking at 48 h (3.65-fold) (**Figure 4D**).

### 3.6 Silencing of *AsCPR* suppresses P450 enzyme activity

**Figure 5:**
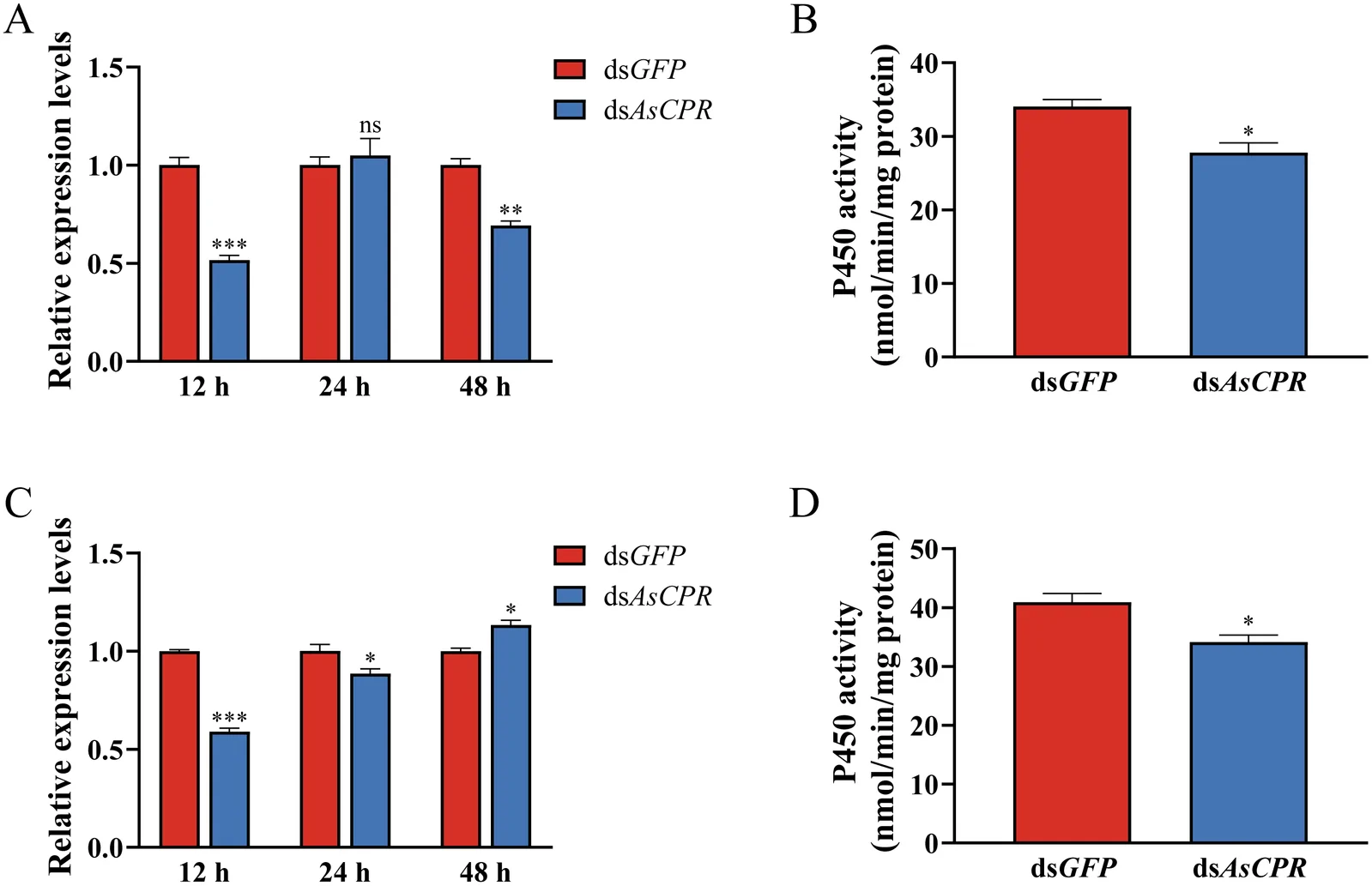
RNAi of the *AsCPR*. Relative expression levels of *AsCPR* in the IM-R **(A)** and LC-R **(C)** strains injected with ds*AsCPR*. The effect of *AsCPR* knockdown on P450 activity in the IM-R **(B)** and LC-R **(D)** strains. Data were shown as mean ± SE (*n* = 3). Asterisks indicate statistically significant differences between the ds*AsCPR*-injected and ds*GFP*-injected groups (Student’s *t*-test, *P < 0.05, **P < 0.01, ***P < 0.001).

To elucidate the role of AsCPR in regulating P450 enzyme activity, P450 activity was measured in *A. spiraecola* following knockdown of *AsCPR*. Compared with the ds*GFP* control group, ds*AsCPR* mediated knockdown of *AsCPR* reached peak silencing at 12 h post-treatment in both IM-R and LC-R strains, with transcript levels reduced by 48.4% and 41.0%, respectively. At the 24 h and 48h posttreatment, the silencing effect was not consistently maintained and largely diminished in both resistant strains (**Figures 5A,C**). As the highest silencing efficiency was observed at 12 h in both strains, this time point was selected for subsequent experiments. The results showed that knockdown of *AsCPR* significantly inhibited P450 enzyme activity, with reductions of approximately 18.4% and 16.6% in the IM-R and LC-R strains, respectively (**Figures 5B, D**).

### 3.7 Silencing of *AsCPR* increases susceptibility of *A. spiraecola* to insecticides

To further investigate the role of *AsCPR* in resistance of *A. spiraecola* to imidacloprid and lambda-cyhalothrin, insecticide susceptibility bioassays were performed on apterous adults following RNAi treatment. The results showed that in the IM-R strain, the LC_50_ value of the ds*GFP* control group was 83.46 mg L^-^¹, whereas that of the ds*CPR* group was 35.39 mg L^-^¹, representing a decrease of 57.6% (**Table 2**). In the LC-R strain, the LC_50_ value of the ds*GFP* group was 839.97 mg L^-^¹, whereas that of the ds*CPR* group was 422.07 mg L^-^¹, corresponding to a decrease of 49.8% (**Table 2**). Collectively, these results indicated that *AsCPR* knockdown increased the susceptibility of *A. spiraecola* to both imidacloprid and lambda-cyhalothrin.

**Table 2:** The toxicity of imidacloprid in the IM-R strain and lambda-cyhalothrin in the LC-R strain of *Aphis spiraecola* after RNAi.

Strains	Treatment	Slope ± SE	LC_50_ (mg L^−1^) (95% CI)	χ2 (*df*)
IM-R	ds*GFP*	2.01 ± 0.16	83.46 (71.87-96.58)	3.11 (3)
	ds*CPR*	1.76 ± 0.15	35.39 (29.96-41.73)	0.70 (3)
LC-R	ds*GFP*	2.09 ± 0.17	839.97 (726.98-965.56)	0.90 (3)
	ds*CPR*	1.96 ± 0.16	422.07 (360.30-491.23)	2.62 (3)

### 3.8 Activity and kinetics of recombinant AsCPR protein

**Figure 6:**
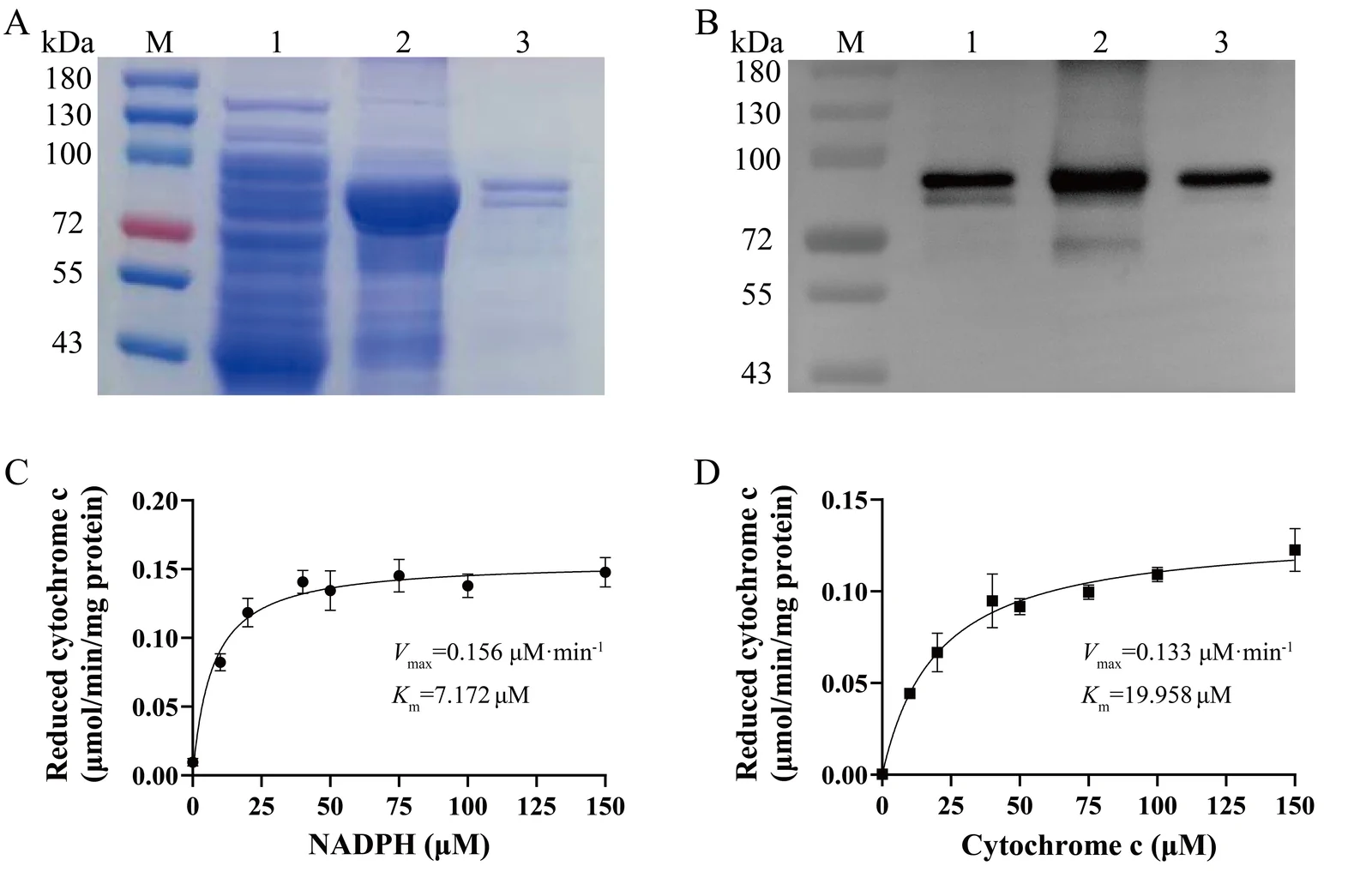
Expression, purification, and kinetic parameter analysis of recombinant AsCPR. **(A)** SDS-PAGE and **(B)** Western blot analyses of AsCPR expression in *E. coli*. Lane M: molecular weight marker; Lane 1: The supernatant induced by IPTG; Lane 2: The pellet induced by IPTG; Lane 3: The purified protein with a His-tag. The kinetic parameters of AsCPR for NADPH **(C)** and cytochrome c **(D)**.

To characterize the enzymatic activity of AsCPR, the recombinant protein was expressed in *E. coli* and purified. SDS-PAGE and WB analyses revealed a single band at approximately 78 kDa (**Figures 6A, B**), matching the predicted molecular weight. The kinetic parameters of AsCPR for NADPH and cytochrome c were determined by fixing one substrate at a final concentration of 100 μM while varying the concentration of the other. AsCPR exhibited Michaelis-Menten kinetic characteristics for both substrates. Specifically, the *V*_max_ and *K*_m_ values for NADPH were 0.156 ± 0.005 μM min^-1^ and 7.172 ± 1.449 μM, respectively (**Figure 6C**), while those for cytochrome c were 0.133 ± 0.005 μM min^-1^ and 19.958 ± 2.761 μM, respectively (**Figure 6D**).

## 4 Discussion

Cytochrome P450s constitute a superfamily of detoxifying enzymes that play pivotal roles in the development of insecticide resistance in pests (Li et al., 2007; Nauen et al., 2022). As the electron donor for P450s, CPR is essential for the catalytic activity of the entire P450 enzyme system (Jing et al., 2018; Huang et al., 2020). In this study, the ORF of *AsCPR* was identified. Sequence alignment and phylogenetic analysis revealed that AsCPR was highly similar to CPRs from other aphids, indicating that the CPR was evolutionarily conserved among insects. Structural prediction showed that AsCPR contained all conserved domains typical of CPRs, including the FAD-, FMN-, and NADP-binding domains. Notably, an N-terminal membrane anchor domain was identified, which targets CPR to the endoplasmic reticulum membrane and is critical for electron transfer to P450s (Laursen et al., 2011). The FMN- and FAD/NADPH-binding domains are connected by a flexible hinge, a structural feature that enables efficient electron transfer by regulating conformational changes in the FMN-binding domain (Laursen et al., 2011). These structural features provided a molecular foundation for understanding the role of AsCPR in insecticide detoxification.

As a vital component of the cytochrome P450 enzyme system, the expression level of *CPR* is closely associated with insecticide resistance in insects (Wang et al., 2016; He et al., 2023). In this study, developmental stagespecific expression analysis revealed that *AsCPR* was expressed at all developmental stages, with the highest expression level detected in apterous adults, suggesting that *AsCPR* may function during the adult stage. Similar expression patterns had been observed in other insects. For instance, in *A. gossypii*, the expression level of *AgCPR* was low in 1st-instar nymphs and peaked in adults (Tang et al., 2023). The transcript of *CPR* was low in the egg and gradually upregulated from 1st-instar nymphs to adults in *Locusta migratoria* (Zhang et al., 2017). Further tissue-specific expression analysis revealed that *AsCPR* was highly expressed in the hemolymph and midgut across all three strains. Consistent with our results, the elevated *CPR* expression in key metabolic tissues, including the midgut, fat body, malpighian tubules and abdomen is often associated with the detoxification of insecticides in various pest insects (Ji et al., 2019; He et al., 2020; Shi et al., 2021). Moreover, the imidacloprid and lambda-cyhalothrin exposure significantly upregulated *AsCPR* expression in both susceptible and resistant strains, suggesting that the overexpression of *AsCPR* contributed to resistance development. Wang X. et al. (2025) found that clothianidin exposure significantly increased *CPR* expression levels in the susceptible and clothianidin-resistant strains of *Bradysia odoriphaga* by 68.6% and 97.9%, respectively. In the lambda-cyhalothrin-resistant strain of *Plutella xylostella*, the expression level of *CPR* could be induced 3.1fold by lambda-cyhalothrin (Chen and Zhang, 2015).

RNAi is a powerful tool for studying gene function in insects (Yang et al., 2021). In the present study, injection of ds*AsCPR* significantly reduced *AsCPR* transcript levels by more than 40% in both the IM-R and LC-R strains, Furthermore, P450 enzyme activity was significantly inhibited and the susceptibility of both strains to imidacloprid and/or lambda-cyhalothrin was substantially enhanced, respectively. Similar results had been reported in other insect species. In *B. odoriphaga*, *CPR* silencing inhibited P450 activity and enhanced susceptibility to clothianidin (Wang X. et al., 2025). Yuan et al. (2021) found that knockdown of *CPR* reduced the detoxification capacity of P450 enzymes, thereby increasing the susceptibility of *Diaphorina citri* against imidacloprid and thiamethoxam. Notably, although some studies did not provide direct evidence of P450 activity assays, CPR knockdown was consistently shown to markedly enhance the pest susceptibility to insecticides. Silencing of *AlCPR* significantly increased the susceptibility of lambda-cyhalothrin-resistant *Apolygus lucorum* to lambda-cyhalothrin (Zhen et al., 2024). Li et al. (2024) reported that knockdown of *TaCPR* significantly enhanced the susceptibility of *Tuta absoluta* larvae against lambda-cyhalothrin. Collectively, these findings suggest that *AsCPR* plays a role in the development of insecticide resistance in *A. spiraecola*. It should be noted that, in the present study, susceptibility bioassays revealed that the LC_50_ value of the ds*GFP* group delivered with SPc was markedly lower than the original LC_50_ values of the resistant strains. This synergistic effect is attributable to SPc-mediated facilitation of insecticide penetration, thereby enhancing insecticide susceptibility (Yan et al., 2019; You et al., 2026).

Heterologous expression systems can efficiently facilitate the study of the specific function of a single gene. In this study, AsCPR was successfully expressed in *E. coli*, and the recombinant protein exhibited electron transfer activity (6.65 nmol/min/mg), indicating that it is a functional enzyme. However, this activity was lower than that of CPR from *Chilo suppressalis* (Liu et al., 2013), *L. migratoria* (Zhang et al., 2023), *Cnaphalocrocis medinalis* (Zhang et al., 2018) and *Anopheles minimus* (Kaewpa et al., 2007), likely due to the low efficiency of prokaryotic systems in producing membrane proteins (Kim et al., 2020). Moreover, the hydrophobic N-terminal anchor domain of CPR tends to misfold and form inclusion bodies, leaving only a small fraction of the protein soluble (Kim et al., 2020; Chen et al., 2023). Notably, CPR from several species had been successfully expressed in truncated forms lacking the N-terminal anchor (Sun et al., 2018; Zhao et al., 2018). Such truncated variants retained the ability to reduce cytochrome c but lost the capacity to interact with P450s (Hayashi et al., 2003). Thus, the full-length AsCPR expressed in this study may facilitate future studies on CPR-P450 interactions. Unfortunately, despite extensive optimization, the specific recombinant P450 proteins could not be obtained, which hindered the *in vitro* assessment of AsCPR and P450-mediated insecticide metabolism.

In conclusion, this study successfully identified the *CPR* gene in *A. spiraecola*, which was highly conserved among aphid species. Sublethal exposure to imidacloprid and/or lambda-cyhalothrin significantly induced the expression of *AsCPR* in susceptible and resistant strains. Silencing *AsCPR* in IM-R and LC-R strains significantly reduced P450s activity and increased the susceptibility of *A. spiraecola* to imidacloprid and lambda-cyhalothrin. Furthermore, AsCPR protein was heterologously expressed, and its electron transfer function was verified through enzymatic characterization. These findings advance our understanding of CPR in P450-mediated insecticide resistance. Future co-expression studies and metabolite analyses are needed to elucidate the insecticide metabolism of the AsCPR/P450 complex.

## Data Availability

The raw sequence data presented in this study have been deposited in the NCBI GenBank database under accession number PZ899843. The data can be accessed via the following link: https://www.ncbi.nlm.nih.gov/nuccore.
