# Supplementary material for: NADPH-cytochrome P450 reductase involved in the multi-resistance of Aphis spiraecola to imidacloprid and lambda-cyhalothrin
Source: Front Physiol. 2026 Sep 11;17:1944804. doi: 10.3389/fphys.2026.1944804 (PMC13612210; doi:10.3389/fphys.2026.1944804)
Supplement: Supplementary file 1 [file Table1.docx]

Supplementary Material

**Table S1** Primers used in the present study.

| Primer name | Application | Sequence (5'-3') |
| --- | --- | --- |
| *AsCPR*-F | qRT-PCR | AGGACTAGCACCATTCCGAG |
| *AsCPR*-R |  | AGGTTGGTCACGCGAAAAATG |
| *β-actin*-F |  | TCGGTATGGGACAAAAAGACA |
| *β-actin*-R |  | GGTCAACAAGATTGGGTGCT |
| *CPR*-Full-F | Full-length amplification | CGCGTATCACGTTCACGGTA |
| *CPR*-Full-R |  | AGAAGCCCACATCTCTTCCA |
| ds*CPR*-F | dsRNA synthesis | TaatacgactcactatagggATCAACCGGTCGTAGTTTGG |
| ds*CPR*-R |  | taatacgactcactatagggTCGTCTCCCAAACCAATTTC |
| ds*GFP*-F |  | taatacgactcactatagggGGAGAAGAACTTTTCACTGG |
| ds*GFP*-R |  | taatacgactcactatagggTGTAGTTCCCGTCATCTTTG |

**Table S2** CPR sequences used for constructing of phylogenetic tree.

| Insect species | GenBank  accession numbers | Insect species | GenBank  accession numbers |
| --- | --- | --- | --- |
| *Achroia grisella* | XP_059054375.1 | *Glossina morsitans* | ADD19306.1 |
| *Acyrthosiphon pisum* | XP_001945312.1 | *Halyomorpha halys* | XP_014294445.1 |
| *Aedes aegypti* | XP_021694731.1 | *Harpegnathos saltator* | NP_001353123.1 |
| *Anopheles funestus* | WXH83406.1 | *Helicoverpa armigera* | ADK25060.1 |
| *Anopheles gambiae* | AAO24765.1 | *Helicoverpa zea* | XP_047020876.1 |
| *Anopheles minimus* | ABL75156.1 | *Laodelphax striatellus* | AHM93010.1 |
| *Anopheles stephensi* | XP_035906466.1 | *Locusta migratoria manilensis* | ANJ65959.1 |
| *Aphis citricidus* | AXR98634.1 | *Mamestra brassicae* | AAR26515.1 |
| *Aphis craccivora* | KAF0767109.1 | *Megachile rotundata* | XP_012145900.1 |
| *Aphis glycines* | KAE9544418.1 | *Musca domestica* | NP_001273818.1 |
| *Aphis gossypii* | XP_050057273.1 | *Musca vetustissima* | XP_061395304.1 |
| *Apis cerana* | XP_061933744.1 | *Myzus persicae* | XP_022170589.1 |
| *Apis dorsata* | XP_031369369.1 | *Nilaparvata lugens* | AHB59865.1 |
| *Apis florea* | NP_001351672.1 | *Ochlerotatus sollicitans* | ACL01092.1 |
| *Bactrocera dorsalis* | NP_001306717.1 | *Odontomachus brunneus* | XP_032667492.1 |
| *Bactrocera oleae* | XP_069966511.1 | *Onthophagus taurus* | XP_022918377.1 |
| *Bemisia tabaci* | XP_018914300.1 | *Ooceraea biroi* | EZA61651.1 |
| *Bombyx mandarina* | XP_028028954.1 | *Orussus abietinus* | NP_001351900.1 |
| *Bombyx mori* | NP_001104834.1 | *Pectinophora gossypiella* | XP_049867648.1 |
| *Camponotus floridanus* | XP_011258274.1 | *Periplaneta americana* | XP_069672074.1 |
| *Chilo suppressalis* | AGM20565.1 | *Plodia interpunctella* | XP_053624999.1 |
| *Cimex lectularius* | NP_001303631.1 | *Plutella xylostella* | NP_001292469.1 |
| *Culex quinquefasciatus* | XP_038114332.1 | *Rhopalosiphum padi* | XP_060835709.1 |
| *Culicoides brevitarsis* | XP_063707533.1 | *Sogatella furcifera* | AHM93009.1 |
| *Dalotia coriaria* | XP_065174334.1 | *Spodoptera exigua* | ADX95746.1 |
| *Danaus plexippus* | OWR41297.1 | *Spodoptera littoralis* | AFP20584.1 |
| *Dendroctonus ponderosae* | XP_019762939.2 | *Spodoptera litura* | XP_022820266.1 |
| *Diachasma alloeum* | XP_015111469.1 | *Thrips palmi* | XP_034251435.1 |
| *Drosophila ananassae* | XP_044572549.1 | *Tribolium castaneum* | XP_064210759.1 |
| *Drosophila melanogaster* | NP_477158.1 | *Triatoma infestans* | ATY75512.1 |
| *Drosophila sechellia* | XP_002038080.1 |  |  |
